# Supplementary material for: Inter-Laboratory Comparison of Metabolite Measurements for Metabolomics Data Integration
Source: Metabolites. 2019 Oct 31;9(11):257. doi: 10.3390/metabo9110257 (PMC6918145; doi:10.3390/metabo9110257)
Supplement: Supplementary file 1 [file metabolites-09-00257-s001.zip › Supplementary Files/Supplementary Material_Figure S1.pdf]

Supplementary Materials

Inter-laboratory comparison of metabolite measurements for metabolomics data integration

Yoshihiro Izumi[1], Fumio Matsuda[2,*], Akiyoshi Hirayama[3], Kazutaka Ikeda[4], Yoshihiro Kita[5], Kanta Horie[6], Daisuke Saigusa[7], Kosuke Saito[8], Yuji Sawada[9], Hiroki Nakanishi[10], Nobuyuki Okahashi[2], Masatomo Takahashi[1], Motonao Nakao[1], Kosuke Hata[1], Yutaro Hoshi[11], Motohiko Morihara[12], Kazuhiro Tanabe[13], Takeshi Bamba[1,*], and Yoshiya Oda[5]

[1] Division of Metabolomics, Medical Institute of Bioregulation, Kyushu University, 3-1-1 Maidashi, Higashi-ku, Fukuoka 812-8582, Japan

[2] Department of Bioinformatic Engineering, Graduate School of Information Science and Technology, Osaka University, 1-5 Yamadaoka, Suita, Osaka 565-0871, Japan

[3] Institute for Advanced Biosciences, Keio University, 246-2 Mizukami, Kakuganji, Tsuruoka, Yamagata 997-0052, Japan

[4] Laboratory for Metabolomics, RIKEN Center for Integrative Medical Sciences, 1-7-22 Suehiro-cho, Tsurumi-Ku, Yokohama, Kanagawa, 230-0045, Japan

[5] Department of Lipidomics, Graduate School of Medicine, The University of Tokyo, 7-3-1 Hongo, Bunkyo-ku, Tokyo 113-0033, Japan

[6] Translational Science, Neurology Business Group, Eisai Co., Ltd., 5-1-3 Tokodai, Tsukuba, Ibaraki 300-2635, Japan

[7] Tohoku Medical Megabank Organization, Tohoku University, 2-1 Seiryo-machi, Aoba-ku, Sendai, Miyagi 980-8573, Japan

[8] Division of Medical Safety Science, National Institute of Health Science, 3-25-26 Tonomachi, Kawasaki-ku, Kawasaki, Kanagawa, 210-9501, Japan

[9] RIKEN Center for Sustainable Resource Science, 1-7-22 Suehiro-cho, Tsurumi-ku, Yokohama, Kanagawa 230-0045, Japan

[10] Research Center for Biosignal, Akita University, 1-1-1 Hondo, Akita-city, Akita 010-8543, Japan

[11] Pharmacokinetic Research Laboratories, Ono Pharmaceutical Co., Ltd., 17-2 Wadai, Tsukuba, Ibaraki 300-4247, Japan

[12] Translational Research Laboratories, Ono Pharmaceutical Co., Ltd., 3-1-1 Sakurai Shimamoto-cho, Mishima-gun, Osaka 618-8585, Japan

[13] Medical Solution Promotion Department, Medical Solution Segment, LSI Medience Corporation, 3-30-1, Shimura, Itabashi-ku, Tokyo 174-8555, Japan

*** Correspondence:** fmatsuda@ist.osaka-u.ac.jp (F.M.); bamba@bioreg.kyushu-u.ac.jp (T.B.); Tel.: +81-66-879-7433 (F.M.); +81-92-642-6171 (T.B.)

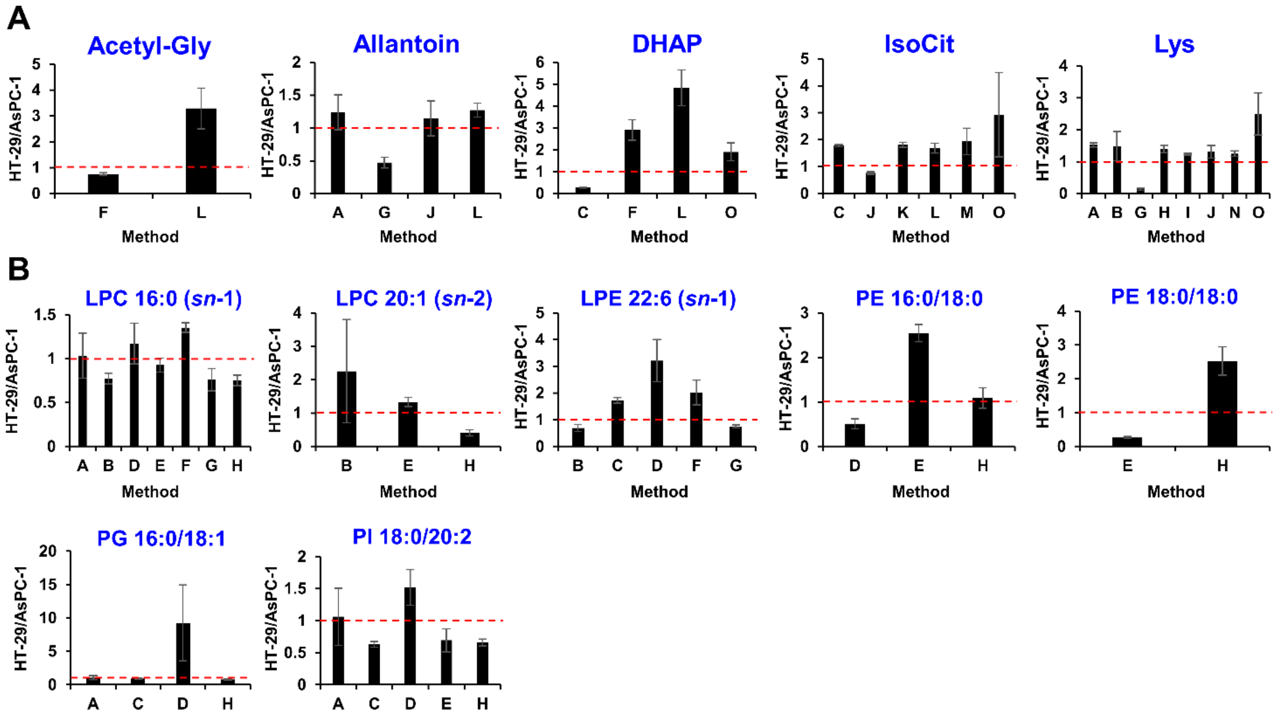

Figure S1. Incomparable relative quantitative values produced from multiple methods. HT-29/AsPc-1 levels of five hydrophilic metabolites (A) and seven hydrophobic metabolites (B) were changed to distinct directions among multiple methods based on two-sided Student's *t*-test ($\alpha = 0.05$) and a relative quantitative value of 1.
